# Genetic and Epigenetic Stability in Rye Seeds under Different Storage Conditions: Ageing and Oxygen Effect

**DOI:** 10.3390/plants9030393

**Published:** 2020-03-23

**Authors:** Michela Pirredda, M. Elena González-Benito, Carmen Martín, Sara Mira

**Affiliations:** Departamento de Biotecnología-Biología Vegetal, Escuela Técnica Superior de Ingeniería Agronómica, Alimentaria y de Biosistemas, Universidad Politécnica de Madrid (UPM), Ciudad Universitaria s/n, 28040 Madrid, Spain; michela.pirredda@upm.es (M.P.); me.gonzalezbenito@upm.es (M.E.G.-B.); mariacarmen.martin@upm.es (C.M.)

**Keywords:** DNA methylation, MSAP, RAPD, *Secale cereale* L., seed ageing, seed storage, storage atmosphere

## Abstract

Seed ageing is a complex process and can be described as the loss of viability or quality with time. It is important to elucidate whether genetic and epigenetic stability is altered in stored seeds and in seedlings produced from them. Non-stored and stored rye seeds at different stages of ageing were compared, as well as the seedlings obtained from them. Seeds were stored at 35 °C and 15% water content, under vacuum or air atmosphere. DNA of seeds and seedlings was isolated at three stages of the deterioration curve: P75 (13 days), P20 (29 days), and P0 (36 days). Genetic stability was assessed by RAPD technique, and epigenetic changes by MSAP markers. While seeds showed genetic stability after storage, the similarity of seedlings obtained from seeds stored for 29 days was lower (95%) when compared to seedlings from control seeds. Epigenetic changes were between 15% and 30% (both de novo methylation and demethylation) in the stored seeds compared to control seeds, with no differences between 13 and 29 days of storage with either air or vacuum atmospheres. In seedlings, epigenetic changes significantly increased with storage time. In conclusion, ageing increased epigenetic instability in both seeds and seedlings, when compared to controls.

## 1. Introduction

Seed ageing is a complex process that can be described as the loss of viability or quality with time. Even if stored under optimal conditions, seeds age losing vigour and viability [1,2]. This can cause important losses for both agronomic production and germplasm banks. 

Seed deterioration during storage increases with either higher seed moisture content and/or temperature [3]. Oxygen availability during storage also influences seed longevity [4,5]. While the exact mechanism causing seed deterioration is still not fully understood, accumulation of reactive oxygen species (ROS) and lipid peroxidation are generally considered major contributors to seed deterioration [6,7,8,9]. ROS can induce geno-toxicity in seeds during storage that would compromise molecular stability, including DNA damage during ageing and the activation of different repair pathways after imbibition [10,11]. Structural damage, single and double strand DNA breaks, or accumulation of point mutations have been reported in ageing seeds [12,13]. Furthermore, DNA fragmentation correlates with seed germination rate [14,15,16]. However, while some of these ageing processes occur, seeds might still be able to germinate. The presence of genetic alterations due to seed ageing in the resulting plants has not been studied in depth.

Cytosine DNA methylation (mC) is a potential heritable epigenetic mark important for regulating gene expression in higher plants and other organisms [17,18]. In plants, as in mammals, DNA methylation has dual roles in defense against invading DNA and transposable elements, and in gene regulation. Methylation of cytosine in vertebrates occur only in CG dinucleotides, while in plants this is more extensive and involves a wider range of methylation sites, being predominantly present at CG dinucleotides [19,20]. Epigenetic changes can modify phenotypes without changing the nucleotide sequence of promoter or coding regions of a gene [21]. These alterations are reversible and can lead to novel and heritable phenotypes [22]. Comparing adult plant tissues to juvenile ones, results suggest that the methylation status of genomic DNA can vary during plant ageing [23,24]. In seeds, epigenetic changes have been detected after drying, both in orthodox and recalcitrant seeds [25,26], and also in seedlings obtained from them [27]. However, epigenetic stability during seed storage and ageing have not been studied extensively, especially regarding what could happen to the next generation. 

The target species of this study is rye (*Secale cereale* L.), an important crop with 4.4 million hectares cultivated in the world in 2016 [28]. Rye was domesticated later than wheat and is able to grow in environments where other cereals cannot, such as cold, semi-arid and high-altitude temperate zones [29]. For these reasons it has been used as a source for improved resistance to pathogens and pests in wheat during more than 50 years [30] and is a species of unquestionable agronomic and economic interest [28]. Moreover, rye seeds are considered to have a medium-short longevity and deteriorate faster than those of wheat [1,31]. This makes rye an interesting model to study species with relatively short longevity during storage.

The main objective of the present study was to investigate changes in the DNA stability and cytosine methylation status during seed storage and ageing. Genetic stability was assessed with Random Amplified Polymorphic DNA (RAPD) technique, and epigenetic changes were studied using Methylation Sensitive Amplified Polymorphism (MSAP) markers. Non-stored and stored rye seeds at different stages of ageing were compared. Also, seedlings obtained from stored seeds were compared to seedlings obtained from control (non-stored) seeds.

## 2. Results

### 2.1. Seed Viability Loss during Storage

**Figure 1:**
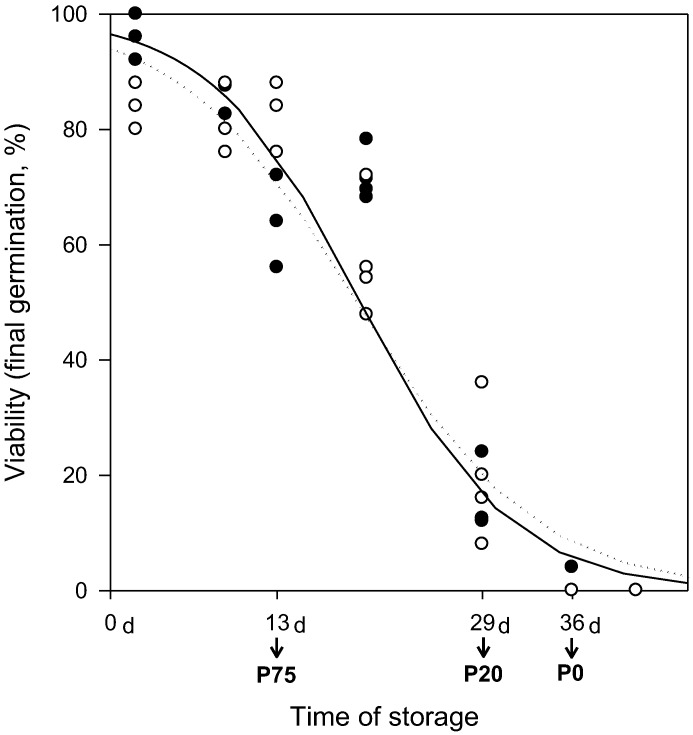
Viability loss of *Secale cereale* seeds during storage at 35 °C, 15% water content (wc) and two different storage atmospheres: air (solid curve, ●) or vacuum (dotted curve, ○). Each data point represents a germination assay in which the percentage of normal seedlings was measured for a particular storage condition and replicate (25 seeds). Data were fitted to a logistic regression model and values for 75, 50, and 20% seed germination (P75, P50, and P20) were calculated (Table 1).

Seed viability decreased with storage time following the characteristic reverse-sigmoidal time course, which starts with an initial asymptomatic stage, characterized as the time for germination to decrease to 75% (P75), and concludes with rapid loss in seed viability, marked by the P20 parameter (Figure 1). Under the storage conditions studied, 35 °C and 15% water content (wc), rye seeds reached P75 by 12–14 days, P20 by 28–29 days and lost their ability to germinate within 35–40 days (P0), showing no differences in ageing kinetics between air or vacuum storage (Table 1, *p* > 0.05).

Genetic and epigenetic stability was evaluated from stored seeds, analyzed immediately after opening the storage bag, and also in imbibed seeds after storage, at different stages of the ageing sigmoidal time course: P75 (13 days), P20 (29 days) and P0 (36 days). In addition, seedlings obtained from stored seeds were also evaluated.

### 2.2. Genetic Stability of Stored Seeds and of Seedlings

The RAPD technique was used to compare DNA profiles of non-stored (control) seeds to stored seeds and also of plants obtained from them, in order to identify changes, using the 10 selected primers. Amplification produced between 7 bands with primers OPF-3, OPF-10, and OPO-5 in seedlings, and 18 bands with primer OPO-20 in seeds. The size of the RAPD amplified DNA fragments ranged from 200 to 2000 base pairs (bp). There were differences between the DNA band pattern of non-stored and of stored seeds, and also in the seedlings obtained from them.

In seeds, 134 amplification fragments were detected in total (Table 2). In stored seeds five primers (OPA-11, OPF-3, OPO-5, OPO-18, and OPO-20) produced eight different polymorphic markers (one per primer except in OPO-18 with which four were detected). In stored-imbibed seeds, three different polymorphic markers were detected, one per each of these primers: OPA-11, OPF-3 and OPO-18. Therefore, few differences were detected in stored seeds, increasing with storage time, both in air and vacuum atmospheres. When these seeds were imbibed, the number of polymorphic markers decreased. The UPGMA cluster analysis of seeds revealed a high percentage of similarity, 98% and 99% in stored and imbibed-stored seeds respectively (data not shown).

**Figure 2:**
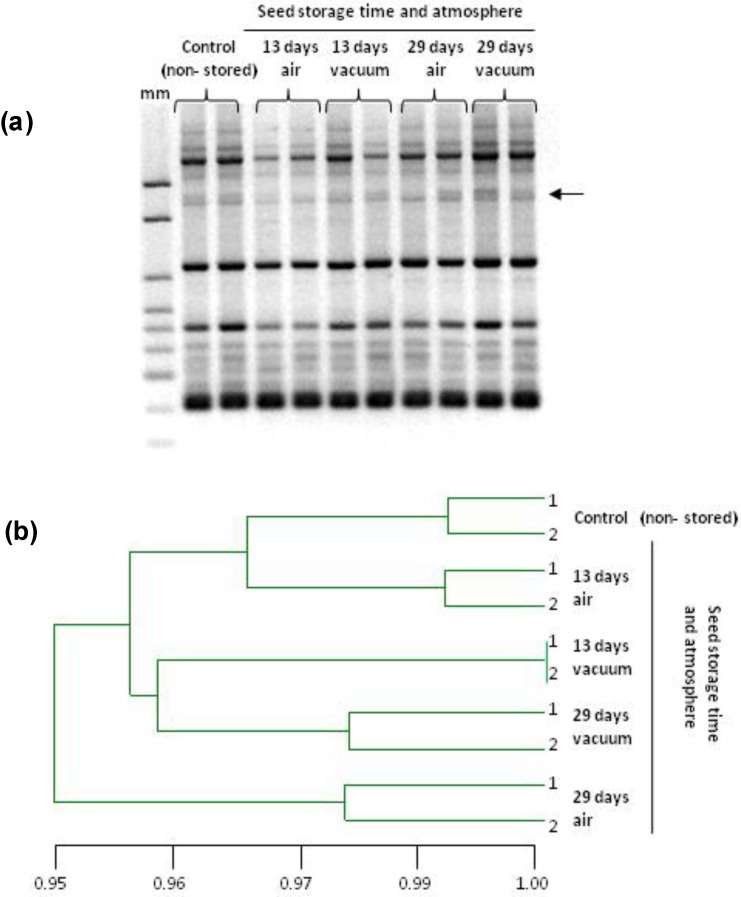
DNA stability in *S. cereale* seedlings obtained from stored seeds: (**a**) Representative RAPD profile of amplification with primer OPO-5 obtained from DNA of seedlings produced from stored and control (non-stored) seeds; (**b**) Dendrogram generated by the UPGMA method using Jaccard’s similarity coefficient, based on RAPD markers. Seeds were stored at 35 °C, 15% wc and two different storage atmospheres: air or vacuum. mm = 100 bp ladder marker. DNA was isolated from two replicates of 10 seedlings per storage condition.

A representative RAPD profile, obtained with primer OPO-5, illustrates the DNA band pattern in seedlings produced from control or stored seeds under air or vacuum (Figure 2). In seedlings, 104 amplification fragments were analyzed (Table 2). The total number of polymorphic bands was nine: one with primers OPF-3, OPO-5, OPO-20; two with OPO-18; and four with OPA-11. After 13 or 29 days of storage, seeds produced seedlings that showed 4 to 5 polymorphic markers. No differences were found in seedlings whether seeds were stored under air or vacuum. The UPGMA cluster analysis of the markers from seedlings revealed that the highest difference was in those produced from 29-day stored seeds under air (95%; Figure 2).

### 2.3. Epigenetic Stability of Stored Seeds and of Seedlings

Epigenetic profiles were generated from stored and stored-imbibed seeds at different ageing stages (13, 29, and 36 days, i.e. P75, P20 and P0, respectively). Analysis revealed a total number of 138 MSAP markers. The methylation status of non-stored (control) seeds was compared before and after imbibition in distilled water for 3 h, and significant differences were found (*p* < 0.05). In non-stored imbibed seeds 24% of MSAP markers showed epigenetic changes respect to seeds before imbibition, with 18% of those being de novo methylation and 6% demethylation. 

**Figure 3:**
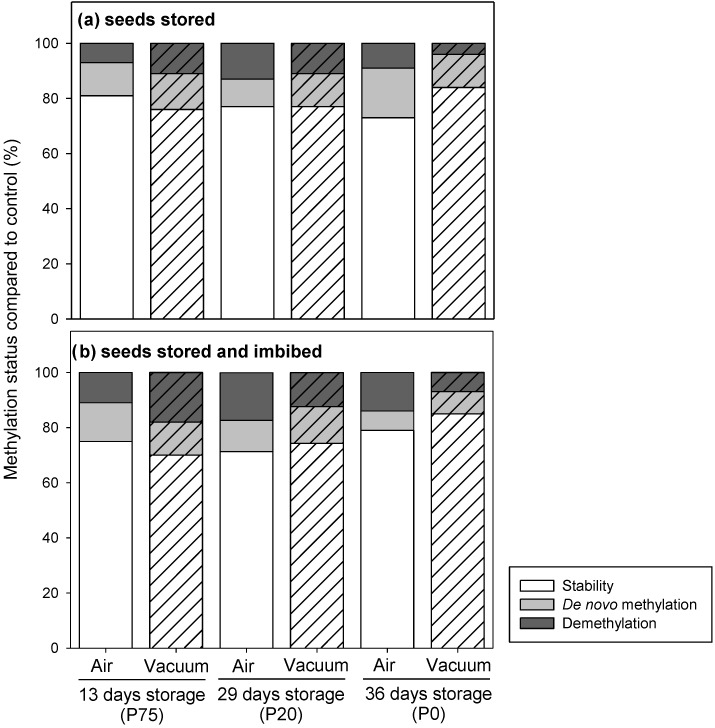
Percentage of stability and epigenetic changes detected in seeds of *Secale cereale*: (**a**) stored seeds; (**b**) stored seeds after imbibition in water. Seeds were stored at 35 °C, 15% wc under two different storage atmospheres: air (filled bars) or vacuum (striped bars). DNA was isolated from two replicates of 50 seeds per storage condition.

Changes in the methylation status (both de novo methylation or demethylation), compared to control seeds, were significantly different among the storage times (*p* < 0.001; Supplementary Material Table S1); the factor atmosphere did not have a significant effect but there was an interaction between storage time and atmosphere. These differences in the epigenetic status compared to control seeds were found from the early asymptomatic stage of seed ageing. After 13 days of storage, seeds had lost very little viability (P75), but epigenetic changes were already detected at similar levels in stored and in stored-imbibed seeds (Figure 3). No significant differences were found between atmospheres of storage (air or vacuum), in stored and stored-imbibed seeds (*p* = 0.28 and *p* = 0.07, respectively, Supplementary Material Table S2). Changes detected in stored seeds were 19 and 24% in air and vacuum, respectively, and 25 and 30% in stored-imbibed seeds (Figure 3). In pair-wise comparisons, no significant differences were found in the epigenetic stability between storing seeds for 13 or 29 days (Figure 3; Supplementary Material Tables S3 and S4). When seeds were stored for 29 days, they were clearly aged with low viability (P20), and 23% epigenetic changes were found for stored seeds with respect to control seeds, and 29–26% (air-vacuum) for stored-imbibed seeds, similar to values obtained at P75. No significant differences were found between the storage atmosphere (air or vacuum), for either stored or stored-imbibed seeds (*p* = 0.67 and *p* = 0.21, Supplementary Material Table S2), which was similar to the results obtained after 13 days.

After 36 days of storage, when the seed lot was completely aged and nonviable (P0), significant differences between air and vacuum storage for both stored and stored-imbibed seeds were found (*p* < 0.05 Supplementary Material Table S2; Figure 3). Percentages of changes detected in stored seeds were 27 and 16% in air and vacuum, respectively, and 21 and 15% in stored-imbibed seeds (Figure 3). 

**Figure 4:**
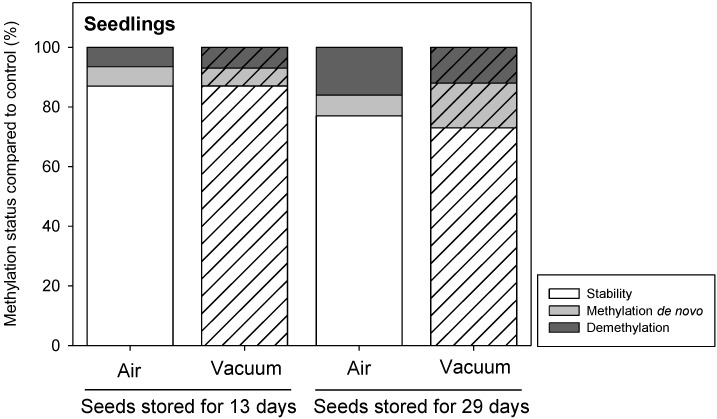
Percentage of stability and epigenetic changes detected in *Secale cereale* seedlings produced from seeds stored at 35 °C, 15% wc under two different storage atmospheres: air (filled bars) or vacuum (striped bars). DNA was isolated from two replicates of 10 seedlings per storage condition.

Epigenetic profiles from seedlings obtained from seeds stored for 13 and 29 days (P75 and P20) showed a total number of 153 MSAP markers. The variations detected increased significantly with storage time (*p* < 0.001; Supplementary Material Table S5). After 13 days of seed storage, 13% of epigenetic changes were detected, independently of the atmosphere condition, increasing to 23–27% (air-vacuum) when seeds were stored for 29 days (Figure 4). No differences were found in seedlings produced from seeds stored in different atmosphere conditions (air or vacuum; *p* = 0.13; Supplementary Material Table S5).

## 3. Discussion

Predicting the onset of seed deterioration during storage is important for seed producers and germplasm conservation. The effect of storage and consequent ageing on DNA stability was studied in *Secale cereale* seeds and regenerated plants at different stages of seed deterioration. Seed ageing follows a sigmoidal pattern in which viability remains relatively constant for an initial phase, followed by an abrupt decline in viability. Genetic and epigenetic changes have barely been studied in stored or aged seeds. 

Genetic stability was high in stored rye seeds; however, some differences were detectable in the plants obtained from them. Similar results were observed in *Mentha aquatica* seeds using the same technique, where seedlings showed lower genetic stability than stored seeds [32]. The RAPD technique has been used to detect seed ageing in several species [16,33,34,35]. Using RAPD analysis, Shatters et al. [36] found differences between non-aged and aged seeds of soybean, by analysing them individually, and also detected polymorphic fragments in plants that were not observed in seed samples. DNA alterations during storage of rye seeds have been reported by analysing fragmentation of nuclear DNA [37]. In the present study, although few differences were detected between stored and stored-imbibed seeds, less polymorphic markers were observed in the latter, which could be related to the activation of DNA reparation mechanism during imbibition in rye, as has been previously described [38]. Nevertheless, some DNA alterations during seed ageing could be irreversible [11,39], which would explain the differences of the produced seedlings. In our study, polymorphism appeared even in those seedlings produced from seeds stored for a short time and with a high viability (P75). The 5% dissimilarity found in seedlings even at the early stage of seed storage should raise concern regarding genetic stability evaluation during seed conservation.

Epigenetic changes in stored seeds were detected, even well before major loss of viability was observed (P75), but they did not significantly increase with storage time nor were influenced by the storage atmosphere. Stress from high temperature and humidity during storage could have induced changes in the methylation state of seeds. In plants, DNA methylation occurs mainly on transposons and other repetitive elements [40], and methylation changes may activate transposable elements and be involved in cytogenetic instability through modification of heterochromatin [41]. Moreover, gene expression regulation by DNA methylation is known to be involved in plant responses to environmental stresses [42]. Variations in the methylation state of stored seeds would result in changes in gene transcription that could affect seed viability. Epigenetic modifications induced by storage stress in seeds might remain in the seedlings after the stress has ceased, as described previously with offspring of asexually reproduced plants [43]. In our study, the longer the seeds were stored, the higher the epigenetic variation in seedlings produced from them was detected. 

Changes in the seed methylation status during storage have been studied before in orthodox and recalcitrant seeds [25,26,27,32]. In the present work, epigenetic changes detected on rye were up to 15–30% in stored seeds, even during the earlier stages of deterioration, and up to 13–27% in seedlings produced from stored seeds. While no increment of variation was associated with storage time in seeds, when the seed lot was still viable (P75 and P20), higher percentages of changes were observed in seedlings after 29 days storage. Similarly, using MSAP technique, stored mint seeds showed an 8% change in epigenetic status (demethylation or de novo methylation) compared to control seeds, and these changes increased to 13% in seedlings produced from aged seeds [32]. In pear, an increase from 3 to 5% in total DNA methylation level of dry seeds (8.8% wc) was found in response to one-year storage [27]. On the other hand, the amount of 5-methylcytosine in genomic DNA decreased with ageing in *Quercus robur* recalcitrant seeds, as viability decreased with time [25]. Recalcitrant seeds suffer viability loss during desiccation. Epigenetic changes associated with drying are highly correlated with viability loss of *Acer pseudoplatanus* recalcitrant seeds but not in *A. platanoides* orthodox seeds [26]. In that study, methylation changes in response to desiccation were not retained in DNA isolated from seedlings, except in seedlings that were derived from strongly desiccated orthodox seeds (3.5% wc). Changes in DNA methylation status after severe desiccation were also detected in orthodox seeds of *Pyrus* (at 2.8% wc), and in seedlings obtained from those seeds [27]. It should be considered that, with the MSAP technique, although the percentages of epigenetic changes detected in seeds after the different treatments (ageing time, air/vacuum, stored/stored-imbibed seeds) were similar, they could correspond to different DNA methylation variations. These differences could explain the fact that seeds showing similar levels of epigenetic changes presented different viability percentages, as the gene regulation processes may not be the same.

In the present work, the effect of oxygen on seed ageing was studied by storing seeds under air or vacuum. Previous studies with dry seeds have reported either neutral or positive effects of anoxic storage on seed longevity [4,5,44,45]. However, negative effects of reduced oxygen were observed upon seed storage at relatively high seed moisture levels [46]. That high seed moisture is approximately above −14 MPa of water potential, as it is the threshold for respiration; providing oxygen increases longevity by sustaining respiration [46]. Our results indicate that neither seed viability parameters nor genetic or epigenetic stability were affected by the atmosphere of storage. The water content of the stored seeds in our study corresponds approximately to −50 MPa (hydration level II) [47], which is below the −14 MPa threshold, which suggests that respiration would not take place and that anoxia would not have negative effects on viability. 

Evaluation of genetic and epigenetic stability in seeds and their derived seedlings should be considered in order to establish optimum storage procedures, even when the consequences of the detected changes cannot be observed in terms of strong viability reduction. During long-term storage, accumulation of genetic changes could affect the genetic profile of the seed lot [48,49]. In addition, as observed in the present study, epigenetic changes may have an important role in the phenotypic result of stored seeds and their seedlings and can be related to viability reduction. A deeper knowledge of the way in which epigenetic changes are acting on the stored seeds is needed in order to understand the mechanisms associated with ageing. Although techniques such as MSAP have shown to be a useful tool as a first step to detect variations [32], further studies are required to establish the best seed storage conditions to avoid rapid loss of viability.

## 4. Materials and Methods 

### 4.1. Seed Storage Experiment

Experiments consisted of adjusting the water content of seeds, enclosing them in hermetically sealed aluminium foil bags, storing them at 35 °C, and sampling periodically to assess seed viability and DNA stability. The experiment was carried out with rye (*Secale cereale* L.) cv. Petkus. Seeds were provided by Agrosa Semillas (Guadalajara, Spain) and stored at 4 °C with silica gel (ca. 7% RH) in darkness until use.

Seed water content was adjusted by equilibrating in the atmosphere of a NaCl saturated solution (75% RH) at 20 °C [50]. Seed aliquots were hermetically sealed into aluminium foil bags (S-156, Valsem Industries SAS, Lachelle, France) with 125 μm thickness of polyester, aluminium and polyethylene layers. Two storage atmosphere conditions were tested: the aluminium bags were closed under normal atmospheric pressure (air storage) or under vacuum (vacuum storage) using an Audionvac VMS 123 (Audion Elektro) vacuum chamber. Aluminium bags were placed at 35 °C for 13, 29 and 36 days of storage.

Seed water content, germination, and DNA stability were checked with two replicates (two bags) per each storage condition (time and atmosphere). Approximately, 300 seeds were stored per bag.

### 4.2. Seed Viability and Water Content Determination

Seed viability loss during storage was determined by germination tests. Germination assays were performed by placing four replicates per storage condition (two replicates per bag). Each replicate consisted of 25 seeds in 15 cm diameter Petri dishes on top of two sheets of filter paper moistened with 10 mL of distilled water. Filter papers were re-wetted regularly with distilled water as required. Incubation conditions were 25 °C with a 16-h photoperiod provided by cool white fluorescent tubes with a photosynthetic photon flux radiance of 35 μmol m^2^ s^−1^. Emergence of the radicle was the criterion for germination. 

In order to test that storage bags were properly sealed, water content (wc) was determined by the low-constant-temperature-oven method [51] and was expressed as percentage on dry weight basis (dw). 

Seed viability loss, as the response to storage time in terms of germination percentage, was modelled using the glm function with a binomial distribution available in the statistical package R (R Core Team, 2015). Times for germination percentage to decrease to 75%, 50% and 20% of maximum germination (i.e. P75, P50, P20) were calculated from the modelled curves for air and vacuum storage conditions using the dose.p function available in R [52]. ANOVA was used to compare viability loss curves with the test statistic for F-tests. 

### 4.3. DNA Extraction and Analysis

DNA from seeds (seed tips) and seedlings was isolated from two replicates per storage condition (one per bag). Replicates for seed DNA extraction consisted of ca. 30 mg obtained from 50 seeds from which the end containing the embryo was excised, in order to eliminate an excess of endosperm. DNA extraction was carried out in seeds immediately after opening the storage bag (hereafter “stored seeds”) or in seeds that after storage were imbibed in distilled water for 3 h (hereafter “stored-imbibed seeds”). Seeds were also germinated to evaluate viability. DNA was isolated from the generated seedlings, when they reached 2 cm of coleoptile (and shoot) length; each replicate consisted of ca. 10 mg obtained from 10 seedlings. In all cases, DNA was extracted using the DNeasy Plant Mini Kit (Qiagen^®^, Verlo, The Netherlands), according to the manufacturer’s instructions. DNA concentration and quality were estimated by spectroscopic analysis. We selected P75 (13 days), P20 (29 days) and P0 (36 days) as stages of deterioration in order to asses genetic and epigenetic stability through storage time. P75 marks approximately the end of the asymptomatic stage of the reverse-sigmoidal viability loss curve, P20 the end of the rapid mortality phase, and at P0 all seeds were dead. Therefore, seedling DNA was extracted after germination of seeds at the P75 and P20 deterioration stages.

### 4.4. Genetic Stability

RAPD analysis was carried out to control DNA stability. A total of 10 random 10-base primers (OPA-11, OPB-15, OPE-19, OPF-3, OPF-10, OPO-5, OPO-6, OPO-7, OPO-18, OPO-20, sequences from Operon Technology) were used to screen the variation on stored and stored-imbibed seeds during ageing and the seedlings produced from them. Two biological replicates were used per storage condition (time and atmosphere). All the reactions were repeated at least twice to monitor the reproducibility of banding patterns and the polymorphism presented.

DNA amplification reactions were performed in a volume of 25 µL containing approximately 10 ng total DNA, 0.8 µM of a single decanucleotide, 5 µL of PCR buffer and 1.25 U MyTaq DNA polymerase (Bioline, London, UK). The PCR amplifications were performed in a SimpliAmpl Thermal Cycler (Thermo Fischer Scientific) using one cycle of 1 min at 95 °C, followed by 35 cycles of 45 s at 92 °C, 1 min at 37 °C and 2 min at 72 °C, and a final cycle of 10 min at 72 °C. RAPD products were loaded onto 1.5 % (*w/v*) agarose gels in 1× TBE buffer at 100 V for 2 h, stained with ethidium bromide (0.5 µg/mL) for 7–10 min and photographed under UV light. The size of the amplified bands was related by reference to the molecular size marker (100 Base- Pair Ladder, GE Healthcare). 

The reproducible RAPD bands were scored in binary characters and a similarity matrix was constructed using the Jaccard’s similarity coefficient, which was further subjected to clustering unweighted pair group method analysis (UPGMA), and a dendrogram was generated. The analysis was performed using the NTSYS-PC software package version 2.

### 4.5. Epigenetic Stability

Epigenetic stability through storage was studied by Methylation Sensitive Amplified Polymorphism (MSAP). The MSAP protocol involving the use of methylation-sensitive isoschizomers HpaII and MspI described by Reyna-Lopez et al. [53] was used, and the HpaII/MspI adapter for the use in plant species was designed according to Xiong et al. [54]. Two biological replicates were used per storage condition (time and atmosphere). 

To identify epigenetic markers, two sets of restriction/ligation reactions were carried out simultaneously. Approximately 500 ng of the extracted DNA was digested with 10 U of EcoRI and 10 U of MspI or 6 U of HpaII, in a final volume of 35 µL containing buffer R-L (10×). The mixture was incubated at 37 °C for 3 h and the reaction stopped by incubation at 65 °C for 5 min; subsequently, samples were cooled for 30–45 min at room temperature. The ligation mixture was carried out adding to the digestion 5 µL of ligation mix [5 pmol EcoRI adaptor, 50 pmol MspI/HpaII adaptor, 10 mM ATP, and 1.2 U T4 DNA ligase (Boehringer) buffer R-L (10×)]. The ligation was incubated for 3 h at 37 °C and overnight at 4 °C. After the incubation, the product of this double reaction was diluted 1:4 to be used as template for the first amplification reaction. Subsequently, these fragments were selectively amplified by fluorescently labelled primers.

The pre-selective PCR reaction was performed in 20 µL containing 30 ng of each primer EcoRI + A and HpaII/Msp I + A, 0.2 mM of each dNTP, 1.5 mM MgCl2, 0.4 U of Taq DNA polymerase (Biotaq, Bioline) and 3 µL of diluted fragments in the 1× PCR buffer provided by the manufacturer (Bioline). The pre-selective amplification was performed with the following profile: 28 cycles of 30 s at 94 °C, 1 min at 60 °C, 1 min at 72 °C. 

Preamplified fragments were diluted tenfold to be used as starting material for the selective amplification. In this amplification, only the EcoRI primer was labelled with FAM fluorochrome; both, the EcoRI and the HpaII/MspI primers contained the same sequences as those used in the preamplification but with three selective nucleotides at the 3′ end for HpaII/MspI primers and two nucleotides for EcoRI primers. Selective amplification was carried out using a total of 3 primer combinations obtained with two EcoRI primers in combination with three HpaII/MspI primers (EcoRI + AC − HpaII/MspI + ATC; EcoRI + AA − HpaII/MspI + AAT/ACT). The PCR reaction was performed in a volume of 20 µL containing 6 ng of labelled EcoRI primer, 30 ng of HpaII/MspI primer, 0.1 mM of each dNTP, 1.5 mM MgCl2, 0.4 U of Taq DNA polymerase, and 5 µL of diluted preamplified DNA in the 1× PCR buffer provided by the manufacturer (Ecogen). The reaction was performed with the following profile: 1 cycle of 30 s at 94 °C, 30 s at 65 °C, 1 min at 72 °C, followed by 12 cycles in which the annealing temperature decreases 0.7 °C per cycle, followed by 23 cycle of 1 min at 94 °C, 30 s at 56 °C and 1 min at 72 °C.

Amplification products were analyzed using an automated ABI3730 sequencer by the company SECUGEN S.L. (Madrid, Spain). The resulted electropherograms were analyzed using GeneMarker v1.90 software (SoftGenetics, LLC, State College, PA, USA) and Peak Scanner V1.0 software. 

MSAP profiles were recorded as 1/0 binary matrix, where 1 indicated the presence and 0 the absence of a given HpaII/MspI marker. In order to identify methylation changes produced during storage, samples of stored seeds were compared to control (non-stored) seeds, and seedlings obtained from stored seeds were compared to those obtained from control seeds. The methylation status of each marker was classified with respect to the control considering three possible events: no change (stability S), demethylation (D) and de novo methylation (M), according to the patterns established by Bardini et al. [55] and Fulneček and Kovařík [56]. The interpretation of MSAP is based on the known restriction enzyme activities at recognition sequences modified by methylation, these data and the corresponding literature can be found at the REBASE website [57]. 

The effect of storage conditions (time and atmosphere) on the methylation status in stored seeds, stored-imbibed seeds and seedlings was analyzed by a multinomial logistic regression model [58]. We used the multinom function from the nnetpackage in R [59]. The response variable was the methylation status (stability S, demethylation D or de novo methylation M) of each sample compared to the control treatment (0 day storage), and the explicative variables were the factors storage time (13, 29 or 36 days) and atmosphere (air or vacuum). Analysis was carried out with the statistical application MAI [60], especially developed to analyze MSAP stability for germplasm conservation purposes. 

## 5. Conclusions

Plants produced from aged seeds showed differences both in genetic and epigenetic stability when compared to control seeds. Rye seeds showed genetic stability after storage, but the similarity of seedlings obtained from stored seeds was lower. In addition, ageing increased epigenetic instability in both seeds and seedlings. Epigenetic changes were detected in stored seeds with no increment of variation associated with storage time in viable samples (P75 and P20), while in seedlings, changes significantly increased with storage time. These epigenetic changes detected in seedlings might have important unknown consequences for germplasm storage procedures. Our data indicate that the quality of stored germplasm should be evaluated both phenotypically and genetically in stored seeds and in the plants obtained after regeneration.

## Figures and Tables

**Table 1:** Longevity parameters and water content of *Secale cereale* seeds stored at 35 °C, after equilibration at 75% RH and 20 °C. Deterioration is expressed as the time for seed germination to decrease to 75, 50, and 20% of initial germination. Data are expressed by mean values ± standard error. *n* = 4 replicates of 25 seeds each.

Atmosphere	WC (% dw)	Seed Longevity Parameters (Days)		
		P75	P50	P20
Air	15.2 ± 0.1	14 ± 2	20 ± 2	28 ± 2
Vacuum	15.1 ± 0.1	12 ± 2	19 ± 2	29 ± 3

**Table 2:** Number of markers obtained from random amplified polymorphic DNA (RAPD) analysis of *Secale cereale* seeds (stored or stored-imbibed) and seedlings produced from them. Seeds were stored at 35 °C, 15% wc under two different storage atmospheres: air or vacuum. DNA was isolated from two replicates of 50 seeds or of 10 seedlings, per storage condition.

Storage Atmosphere	Number of Polymorphic Markers							
	Stored Seeds			Stored-Imbibed Seeds			Seedlings	
	Storage Time (Days)			Storage Time (Days)			Seed Storage Time (Days)	
	13 (P75)	29 (P20)	36 (P0)	13 (P75)	29 (P20)	36 (P0)	13 (P75)	29 (P20)
**Air**	3	4	5	0	1	1	4	5
**Vacuum**	0	2	3	2	1	1	4	5
**Total**	134	134	134	134	134	134	104	104

## Supplementary Materials

The following are available online at https://www.mdpi.com/2223-7747/9/3/393/s1, Table S1: Likelihood-ratio test (G2) results of the percentages of methylation status changes (Figure 3) determined by MSAP markers in stored and stored-imbibed seeds of *Secale cereale*, Table S2: Likelihood-ratio test (G2) results of the percentages of methylation status changes (Figure 3) determined by MSAP markers in stored seeds of *Secale cereale*: pair-wise comparison between storage atmosphere (air or vacuum), for each storage time, Table S3: Likelihood-ratio test (G2) results of the percentages of methylation status changes (Figure 3a) determined by MSAP markers in stored seeds of *Secale cereale*: pair-wise comparison between storage times, Table S4: Likelihood-ratio test (G2) results of the percentages of methylation status changes (Figure 3b) determined by MSAP markers in stored-imbibed seeds of *Secale cereale*: pair-wise comparison between storage times, Table S5: Likelihood-ratio test (G2) results of the percentages of methylation status changes (Figure 4) determined by MSAP markers in seedlings of *Secale cereale* produced from seeds stored at 35 °C, 15% wc with different storage atmospheres (air or vacuum) and times (13 and 29 days).
