# Supplementary material for: Genetic and Epigenetic Stability in Rye Seeds under Different Storage Conditions: Ageing and Oxygen Effect
Source: Plants (Basel). 2020 Mar 23;9(3):393. doi: 10.3390/plants9030393 (PMC7154831; doi:10.3390/plants9030393)
Supplement: Supplementary file 1 [file plants-09-00393-s001.pdf]

Supplementary material: Statistical analyses

Table S1. Likelihood-ratio test (G2) results of the percentages of methylation status changes (Figure 3) determined by MSAP markers in stored and stored-imbibed seeds of *Secale cereale*. Seeds were stored at 35 °C, 15% wc under two different storage atmospheres (air or vacuum) and times (13, 29 or 36 days).

		Stored Seeds		Stored-Imbibed Seeds	
Factors	Df	G2	P-value	G2	P-value
Storage time	4	13.20	0.01	19.29	<0.001
Atmosphere	2	1.22	0.54	1.35	0.51
S. time x Atmosphere	4	14.04	0.01	15.41	<0.001

Table S2. Likelihood-ratio test (G2) results of the percentages of methylation status changes (Figure 3) determined by MSAP markers in stored seeds of *Secale cereale*: pair-wise comparison between storage atmosphere (air or vacuum), for each storage time.

Storage Time	Factor: Atmosphere				
		Stored Seeds		Stored-Imbibed Seeds	
	Df	G2	P-value	G2	P-value
13 days (P75)	2	2.54	0.28	5.34	0.07
29 days (P20)	2	0.80	0.67	3.09	0.21
36 days (P0)	2	11.93	< 0.001	8.33	0.02

Table S3. Likelihood-ratio test (G2) results of the percentages of methylation status changes (Figure 3a) determined by MSAP markers in stored seeds of *Secale cereale*: pair-wise comparison between storage times.

Pair-wise comparison	Df	Air		Vacuum	
		G2	P-value	G2	P-value
**13 days vs 29 days**	2	4.69	0.1	0.07	0.97
13 days vs 36 days	2	6.43	0.04	11.48	< 0.001
29 days vs 36 days	2	8.31	0.02	11.29	< 0.001

Table S4. Likelihood-ratio test (G2) results of the percentages of methylation status changes (Figure 3b) determined by MSAP markers in stored-imbibed seeds of *Secale cereale*: pair-wise comparison between storage times.

Pair-Wise Comparison	Df	Air		Vacuum	
		G2	P-value	G2	P-value
13 days vs 29 days	2	4.65	0.10	3.66	0.16
13 days vs 36 days	2	8.93	0.01	20.10	< 0.001
29 days vs 36 days	2	6.25	0.04	10.19	0.01

Table S5. Likelihood-ratio test (G2) results of the percentages of methylation status changes (Figure. 4) determined by MSAP markers in seedlings of *Secale cereale* produced from seeds stored at 35 °C, 15% wc with different storage atmospheres (air or vacuum) and times (13 and 29 days).

**Factors**	**Df**	**G2**	**P-value**
Storage time	2	20.31	< 0.001
Atmosphere	2	4.15	0.13
S. time x Atmosphere	2	3.64	0.16
